# Clinical Pharmacokinetics and Safety of a 10% Aminolevulinic Acid Hydrochloride Nanoemulsion Gel (BF‐200 ALA) in Photodynamic Therapy of Patients Extensively Affected With Actinic Keratosis: Results of 2 Maximal Usage Pharmacokinetic Trials

**DOI:** 10.1002/cpdd.1023

**Published:** 2021-10-11

**Authors:** Ben Novak, Janet DuBois, Osama Chahrour, Tamara Papusha, Stefan Hirt, Thomas Philippi, Corinna Zogel, Katharina Osenberg, Beate Schmitz, Hermann Lübbert

**Affiliations:** ^1^ Biofrontera Bioscience GmbH Leverkusen Germany; ^2^ DermResearch Inc. Austin Texas USA; ^3^ ACM Bioanalytical Services York UK; ^4^ CRS Clinical Research Services Moenchengladbach GmbH Moenchengladbach Germany; ^5^ Nuvisan GmbH Neu‐Ulm Germany; ^6^ CRS Clinical Research Services Mannheim GmbH Mannheim Germany

**Keywords:** aminolevulinic acid, BF‐200 ALA, BF‐RhodoLED, maximal usage pharmacokinetic trial, nanoemulsion, protoporphyrin IX

## Abstract

The nanoemulsion‐based 10% aminolevulinic acid (ALA) hydrochloride gel BF‐200 ALA optimizes epidermal penetration of its active ingredient and is approved for topical photodynamic therapy (PDT) for the treatment of actinic keratosis in the United States and Europe. To characterize systemic absorption from dermal application during PDT, ALA and its key active metabolite protoporphyrin IX (PpIX) were analyzed in 2 maximal usage pharmacokinetic trials (MUsT) in patients severely affected with actinic keratosis. The primary objective of both MUsTs was to assess baseline‐adjusted plasma concentration–time curves for ALA and PpIX after a single PDT treatment applying either 2 g (1 tube) of BF‐200 ALA on the face (MUsT‐1) or applying 6 g (3 tubes) of BF‐200 ALA on the face/scalp or body periphery (MUsT‐2), to 20 or 60 cm^2^, respectively. All PDTs were performed using red light at around 635 nm wavelength. Safety and tolerability were documented along with pharmacokinetics. In both MUsTs, ALA plasma concentrations were transiently increased to a maximum concentration at about 2.5 to 3.3 times above endogenous baseline with time to maximum concentration at ≈3 hours after dosing. Plasma levels subsequently returned to baseline within 10 hours after dosing. Overall baseline‐adjusted mean area under the baseline‐adjusted plasma concentration‐time curve from time zero to the last sampling time point at which the concentration was at or above the lower limit of quantification ranged from 142.8 to 146.2, indicating that a similar, minor fraction of topical ALA is systemically absorbed under both dosing regimens. Systemic PpIX exposure after administration of either dose of BF‐200 ALA was equally minimal. Application site skin reactions were treatment area size‐related, albeit transient and consistent with the known safety profile of BF‐200 ALA.

Actinic keratoses (AKs) are atypical proliferations of epidermal keratinocytes that are regarded as precancerous and typically develop because of chronic exposure to ultraviolet radiation.^1^ AK is the most common dermatologic diagnosis in patients ≥45 years of age in the United States.^2^, ^3^ AK represents an early stage of a malignant condition (carcinoma in situ) that can progress to squamous cell carcinoma (SCC). The risk for progression to invasive SCC for a specific AK lesion ranges between 0.025% and 16%.^4^ Without intervention, resulting SCC may subsequently lead to significant health care expenditure, patient morbidity, and patient mortality.^3^ AK lesions are typically embedded in areas of photo‐damaged skin where different stages of AK may coexist, including subclinical (nonvisible, nonpalpable) lesions.^5^ Recent clinical guidelines for the treatment of AK recognize the importance of field‐directed treatments to provide long‐lasting disease remission and prevent disease recurrence and eventually the development of invasive SCC.^6^, ^7^, ^8^, ^9^

Photodynamic therapy (PDT) using topically applied prodrugs of the endogenous, photosensitizing heme precursor protoporphyrin IX (PpIX), such as 5‐aminolevulinic acid (ALA), displays high selectivity and efficacy and is therefore recommended in international guidelines for field‐directed treatment of AK.^6^, ^10^, ^11^ Biofrontera's (BF; Leverkusen, Germany) proprietary nanoemulsion BF‐200 significantly enhances the stability of the active ingredient and improves epidermal penetration.^12^, ^13^, ^14^ Because of this, the percentage of ALA in BF‐200 ALA (Ameluz) could be reduced to 7.8% (equivalent to 10% ALA hydrochloride), half that of other marketed ALA or methyl‐ALA formulations.^12^ Clinical efficacy and safety of BF‐200 ALA in combination with red‐light PDT for the treatment of mild to moderate AK on the face and scalp were examined in 1 dose‐finding study and 3 confirmatory studies encompassing a total of 885 randomized patients comprising 412 patients exposed to BF‐200 ALA (7.8% ALA) in combination with red light.^15^, ^16^, ^17^ Subsequently, 1 confirmatory intraindividual study was performed for the assessment of clinical efficacy and safety of BF‐200 ALA for the treatment of AKs located on extremities or trunk/neck.^18^ In these pivotal clinical trials, complete clearance of patients 12 weeks after the first PDT with BF‐200 ALA was shown to be up to 61.8% and up to 90.9% after a maximum of 2 PDTs.^15^, ^16^, ^17^ Another pivotal study demonstrated the efficacy and safety of PDT with BF‐200 ALA on superficial and nodular basal cell carcinomas.^19^ For mild to moderate AK on the face and scalp, another pivotal trial demonstrated high efficacy in PDT with natural daylight.^20^ BF‐200 ALA is used in the routine clinical treatment of mild to moderate AK and field cancerization with both red light and natural daylight and for the treatment of superficial and nodular basal cell carcinoma with red light in Europe.^17^, ^18^, ^19^, ^20^ In the United States, it is applied in combination with the red‐light BF‐RhodoLED lamp for the lesion‐ and field‐directed PDT of AK of mild to moderate severity on the face and scalp.^15^, ^16^, ^17^ Current standards for the development of topically applied drugs demand the characterization of systemic absorption upon dermal application within the framework of maximal usage trials (MUsTs).^21^ This was done for BF‐200 ALA in 2 pharmacokinetic studies in individuals extensively affected with AK, using either a single 2‐g dose of BF‐200 ALA gel on the face, or 3 simultaneous 2‐g doses for extended areas on the face, scalp, or body periphery (neck/trunk/extremities). The relevance of treating peripheral body regions results from the fact that skin areas presenting with chronic actinic damage almost exclusively occur on the sun terraces of the body, which include not only the head and neck but also, for example, the upper limbs and décolleté.^22^ In addition, expansion of fields with chronic actinic sun damage is likely to exceed the size of the current treatment area of 20 cm^2^ for BF‐200 ALA,^23^, ^24^ particularly when also taking AK treatment in the periphery into consideration. This article reports the results for ALA and PpIX pharmacokinetics in plasma along with safety and tolerability under maximal use conditions from both trials.

## Methods

The 2 MUsTs were conducted between 2013 and 2020 in accordance with the Declaration of Helsinki, guidelines of Good Clinical Practice, and other relevant regulatory guidelines. Both studies were approved by competent institutional review boards/independent ethics committees before implementation. For MUsT‐1, the ethics committee was of the Medical Association North Rhine, in Duesseldorf, Germany, and for MUsT‐2, it was the central institutional review board Advarra, in Columbia, Maryland. All participants provided written informed consent before study enrollment.

### Study Design

#### Maximal Usage Pharmacokinetic Trial 1

**Figure 1:**
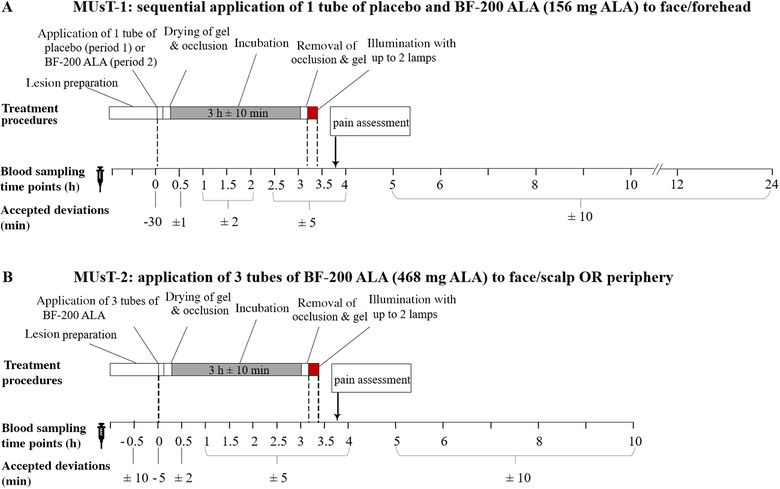
Blood sampling and intervention schedule on treatment day. Blood sampling time points are specified in hours relating to the time point of BF‐200 ALA application. Time point 0 is defined as the start of the application of BF‐200 ALA. (A) MUsT‐1 was designed as a fixed‐sequence trial, comparing placebo and BF‐200 ALA sequentially in the same cohort with an identical intervention protocol. Patients were treated on the face or forehead with 2 g BF‐200 ALA (156 mg ALA) and illuminated after 3 hours of incubation with up to 2 lamps. (B) MUsT‐2 was designed as an open‐label trial, patients were treated on either the face/scalp or body periphery with 6 g BF‐200 ALA (468 mg ALA) and illuminated after 3 hours of incubation with up to 2 lamps. ALA, 5‐aminolevulinic acid; MUsT, maximal usage pharmacokinetic trial.

The first MUsT (ALA‐AK‐CT006, EudraCT No. 2013 000339 28) was a single center, nonrandomized, open‐label, placebo‐controlled, fixed‐sequence study to evaluate the pharmacokinetics of ALA and PpIX in the plasma of patients with AK following topical application of 2 g of BF‐200 ALA (containing 78 mg/g ALA) under maximal use conditions when using PDT. It was conducted in 1clinical phase I unit at CRS Clinical Research Service, Moenchengladbach, Germany. In this study, eligible patients were aged 18 to 85 years and presented with at least 10 lesions of AK of mild to moderate intensity (Olsen grade 1 and 2)^25^ in a total treatment area of 20 cm^2^ on the face or forehead. Treatment fields were allowed to be discontinuous but must have been within 2 illumination areas of the PDT lamp (6 cm × 16 cm each). A fixed‐sequence design was chosen, in which all patients received PDT with placebo (period 1) followed by PDT with BF‐200 ALA (period 2) with an interim washout period of 7 days. Both placebo and BF‐200 ALA were applied at a thickness of 1 mm in a quantity of 2 g to an area of 20 cm^2^ on the face or forehead. For BF‐200 ALA, this resulted in a total drug dose of 156 mg. Treatments fields were allowed to be discontinuous but must have been within 2 illumination areas of the PDT lamp (6 cm × 16 cm each). The study medication was incubated for 3 hours under an occlusive, light‐blocking dressing. Thereafter, the occlusion and remaining gel were removed, and a PDT illumination was conducted, delivering a total light dose of 37 J/cm^2^ at a peak wavelength of 635 nm with 1 or, when needed, 2 PDT lamps simultaneously. On both treatment days, a total of 15 blood samples were taken per patient. Their time points in relation to BF‐200 ALA application were: 0 (predose), 0.5, 1, 1.5, 2, 2.5, 3, 3.5, 4, 5, 6, 8, and 10 hours. A graphical overview of the treatment and sampling procedures in MUsT‐1 is given in Figure 1A.

#### Maximal Usage Pharmacokinetic Trial 2

The second MUsT (ALA‐AK‐CT015, NCT04319159) was a single‐center, nonrandomized, open‐label phase I study to evaluate the pharmacokinetics of ALA and PpIX in the plasma of patients with AK after topical application of 3 tubes of BF‐200 ALA 10% gel for PDT under maximal use conditions. It was conducted in 1 clinical phase I unit at DermResearch, Inc., Austin, Texas. In this study, eligible patients were 18 to 85 years of age, and presented with at least 12 clinically confirmed mild to severe AK lesions (according to Olsen et al^25^) on either the face/scalp or the neck/trunk/extremities, within treatment fields of about 60 cm^2^ in total. Treatments fields were allowed to be discontinuous but must have been within 2 illumination areas of the PDT lamp (6 cm × 16 cm each). This study was designed open‐label as the use of a placebo arm was considered unnecessary based on the previous observation from MUsT‐1 that plasma concentrations of ALA and PpIX are not subject to circadian rhythm. The PDT procedure (preparation of treatment field[s], drug application, incubation, illumination) was performed similar to MUsT‐1 with the exceptions that 3 tubes of BF‐200 ALA (totaling a dose of 468 mg ALA) were applied per patient in an extended skin area and the treatment was performed on the face/scalp or in the periphery (neck/trunk/extremities) with 1 or, when needed, 2 PDT lamps simultaneously. The number of blood samples taken for pharmacokinetics as well as the time points of blood sampling were determined on the basis of the results of the previous MUsT‐1, taking into account expected time to reach maximum concentration (t_max_) and apparent terminal half‐life (t_1/2_), thus avoiding unnecessary blood sampling and shortening the time over which blood samples were collected. On treatment days, a total of 14 blood samples were taken per patient. Their time points in relation to BF‐200 ALA application were –0.5, 0 (predose), 0.5, 1, 1.5, 2, 2.5, 3, 3.5, 4, 5, 6, 8, and 10 hours. An additional predose sample was taken at the screening visit, such that 3 predose samples per patient could be analyzed to establish individual baseline plasma levels of ALA and PpIX. A graphical overview of the treatment and sampling procedures in MUsT‐2 is given in Figure 1B.

### Bioanalytical Methodology

The concentrations of ALA and PpIX in plasma samples were measured using internally standardized liquid chromatography–tandem mass spectrometry (LC‐MS/MS) methods. The methods were developed and validated according to current international standards.^26^ The analytical methods differed slightly between studies and are detailed per study below, along with specific core information. Per blood draw for bioanalysis, 5 to 10 mL of blood were collected in lithium heparin tubes, and samples were handled under appropriate red or yellow light conditions to avoid photobleaching of PpIX. Samples were stored on ice until centrifugation, plasma samples were stored frozen until shipment, shipped on dry ice under temperature control, and handled under the same appropriate light conditions at the bioanalysis facilities. Since both ALA and PpIX are endogenous compounds, drug‐free matrix is not available, and appropriate alternative matrices were prepared (see below). For in‐process assessment of methodical quality of the sample analyses in both studies, intrabatch precision (expressed as coefficient of variation) and intrabatch accuracy were measured over all calibration standards and quality control (QC) samples of the respective batches and analyzed globally for all runs. A summary of the performance of the bioanalytical methods is given in the results section and in Table S3.

#### Maximal Usage Pharmacokinetic Trial 1

In MUsT‐1, for the determination of ALA and PpIX in plasma, a total of 360 blood samples were shipped to CRS laboratory (30 samples per period from each of the 12 patients). All collected samples were analyzed and had valid results. The lower limits of quantification (LLOQ) of ALA and PpIX in plasma were 1.000 ng/mL. ALA was determined by an internally standardized LC‐MS/MS method using atmospheric pressure chemical ionization in the positive ion mode. Buffer at pH 6 was used as artificial matrix. Analyte PpIX was determined by an internally standardized LC‐MS/MS method using electrospray ionization in the positive ion mode. Bovine serum albumin (fraction V) was used as artificial matrix. Before detection in multiple reaction monitoring on a mass spectrometer, analyte and internal standard were separated on an analytical narrow bore column. An overview of instrument settings and equipment is given in Table S1. Stock and working solutions for both analytes were prepared independently for calibration standards and quality control samples. Working solutions for ALA were prepared freshly by spiking buffer pH 6 with ALA in deionized water. The quantification of ALA in human plasma was achieved via external calibration using 5‐ALA‐^13^C_2_, ^15^N HCl as an internal standard. Calibration standards were prepared by spiking buffer pH 6 as replacement matrix with ALA at 8 concentration levels ranging from 1.000 ng/mL through 100.000 ng/mL. Before analysis, ALA was derivatized using a method described below (see MUsT‐2). For PpIX, working solutions were prepared by spiking methanol with PpIX in methanol/dimethylformamide (1/1). Midalzolam‐D4 was used as an internal standard, and calibration standards were prepared by spiking blank phosphate buffered saline in bovine serum albumin, fraction V, as artificial matrix with PpIX in methanol at 8 concentration levels ranging from 1.000 ng/mL through 100.000 ng/mL. For ALA, QC samples were prepared by spiking buffer pH 6 with 3.000 ng/mL (low‐QC), and by spiking blank plasma diluted by a factor of 4 with 33.654 ng/mL (mid‐QC) and 78.654 ng/mL (high‐QC), respectively. Additionally, bio‐QC, which represents the endogenous concentration, was prepared by diluting blank plasma by a factor of 4. The sum of the mean diluted endogenous background concentrations plus the respective spiked concentrations were used as theoretical concentrations for the mid‐ and high‐QC. The mean diluted endogenous background is used as theoretical concentration for the bio‐QC. The mean endogenous background concentration was 14.619 ng/mL. Since plasma is diluted by a factor of 4 with buffer pH 6, the diluted concentration is then calculated to be 3.654 ng/mL. For PpIX, as endogenous levels in blank plasma could not be determined with sufficient certainty, spiked concentrations were regarded as theoretical concentrations. QC samples were prepared by spiking blank bovine serum albumin as artificial matrix with PpIX in methanol. QC samples were prepared at 3 concentration levels: 3.000 ng/mL (low‐QC), 23.000 ng/mL (mid‐QC), and 80.000 ng/mL (high‐QC). Analysis was performed as a single determination. Samples were ordered according to their expected concentration to avoid carryover.

#### Maximal Usage Pharmacokinetic Trial 2

A total of 960 plasma samples were shipped to ACM Bioanalytical Services for the determination of ALA (480 samples) and PpIX (480 samples). All collected samples were analyzed and had valid results, with 1 single exception: as 1 of the samples for PpIX analysis had not been adequately protected from light during transport, it had to be excluded from final analysis. Buffer (citric acid/sodium hydroxide, pH 6) was used as an artificial matrix for the calibration standards, LLOQ, and low‐QC. Calibration standards were prepared at 0.00 (blank), 1.00, 2.50, 5.00, 12.5, 25.0, 50.0, 80.0, and 100 ng/mL, from the ALA reference material. LLOQ and low‐QC samples were prepared at 1.00 and 3.00 ng/mL, respectively. Mid‐ and high‐QC samples were prepared by spiking ALA into plasma to give concentration increases of 120 and 300 ng/mL, respectively. Since plasma samples were diluted 4 times before extraction, the diluted mid‐ and high‐QC measured concentrations were 30.0 and 75.0 ng/mL plus the diluted plasma endogenous content determined from the bio‐QC. The quantification of ALA in human plasma was achieved via external calibration using 5‐ALA‐^13^C_2_, ^15^N HCl as an internal standard. ALA was extracted from samples (50 μL) of diluted human plasma (containing lithium heparin as an anticoagulant) or buffer pH 6 by protein precipitation using methanol (150 μL) and then 100 μL of the supernatant was transferred to a new well and evaporated to dryness under nitrogen. The analyte was derivatized by adding 50 μL 1‐butanol:HCl (2:1 v/v), heating at 65°C for 30 minutes, and then evaporating until dry. The derivatization product 5‐aminolevulinic acid butyl ester was reconstituted in 100 μL reconstitution solvent (87 mL water, 0.5 mL formic acid, 12.5 mL acetonitrile) for quantitative determination by LC‐MS/MS with multiple reaction monitoring and atmospheric pressure chemical ionization in the positive ion mode. An overview of instrument settings and equipment is given in Table S2. For analysis of ALA, a batch usually contained 5 replicates of the control matrix used to establish the endogenous amount of ALA present within the matrix to correct the concentration of the QC samples prepared using that matrix.

Plasma devoid of endogenous PpIX and was used to prepare calibration standards using PpIX reference material at 0.00 (blank), 1.00, 2.50, 5.00, 12.5, 25.0, 50.0, 80.0, and 100 ng/mL. QC samples containing PpIX in depleted plasma were prepared at 1.00, 3.00, 30.0, and 75.0 ng/mL. The quantification of PpIX in human plasma was achieved via external calibration using PpIX D6 as an internal standard. PpIX was extracted from samples (100 μL) of plasma by protein precipitation. The analyte was extracted using methanol:acetonitrile (50:50, v/v) in 0.1% formic acid. After centrifugation, the supernatant was transferred to a second 96‐well plate for quantitative determination using LC‐MS/MS with multiple reaction monitoring and electrospray ionization in the positive ion mode. An overview of instrument settings and equipment is given in Table S2.

All samples for a given patient were analyzed together in a single batch except when samples had to be reanalyzed. A batch, at a minimum, consisted of duplicate sets of calibration standards and duplicate low‐, med‐, and high‐QC samples. Analysis batches that contained study samples that were diluted into range also contained a QC sample that was similarly diluted.

### Safety Assessments

In both MUsTs, collection of adverse events (AEs) started at signing informed consent until the end of the study. AEs were discriminated as pretreatment adverse events and treatment‐emergent adverse events (TEAEs). Both application site pain and application site reactions were collected from the outset of treatment. In MUsT‐1, this assessment encompassed local and overall tolerability, including pain, graded by the investigator on a 4‐point scale (absent, mild, moderate, severe). In this study, application site pain and skin reactions in the treatment area triggered by the PDT were documented but not considered as AEs. Pain was documented after the illumination. A pretreatment with 1 g acetaminophen 1 hour before illumination was considered at the discretion of the study physician. Application site skin reactions were collected on days 1 and 7 after treatment and allocated to the predefined terms: burning, dryness, edema, erosion, erythema, and itching. In MUsT‐2, patients assessed the experienced pain during PDT using an 11‐point numeric rating scale ranging from 0 (no pain at all) to 10 (worst possible pain). This score reflected the patient's respective maximum pain during treatment. In this study, skin reactions occurring in the treatment field(s) after starting PDT treatment were subclassified into the more specific categories application site discomfort and application site skin reactions and were documented on the treatment day and on days 7 and 28 after treatment. The assessment on day 7 was via phone call and captured the patient‐reported events only. Application site discomfort encompassed events such as “burning,” “pain,” “itching,” “stinging,” and others. Intensity was assigned to 1 of 4 categories ranging from “none” to “severe” based on the patient's report. Application site skin reactions, like “erythema,” “edema,” “induration,” “vesicles,” and others were assessed by the investigator on the treatment day and at the subsequent visit. Intensity assessment followed the discomfort intensity grading. AEs were tabulated by the Medical Dictionary for Regulatory Activities preferred term and system organ class. In addition to this, routine laboratory testing from blood and urine was performed before and after treatment. The safety end points were analyzed descriptively and in an exploratory way.

### Pharmacokinetic/Statistical Analysis

Both ALA and PpIX are endogenous molecules, naturally occurring in human plasma. Hence, bioanalytical data were corrected by each patient's individual baseline and the primary end point analyses focused on baseline‐corrected concentration‐vs‐time profiles and baseline‐corrected PK parameters. Baseline was established from one predose plasma sample (MUsT‐1) or the mean of 3 predose plasma samples (MUsT‐2; see above). Patient‐specific baseline values were used for baseline adjustment in each study. Values less than the LLOQ and negative values after baseline‐adjustment were set to 0 and disregarded for the calculation of geometric statistics and on the logarithmic scale. In MUsT‐1, data were analyzed by period (placebo and BF‐200 ALA). In MUsT‐2, data were analyzed overall and according to strata (treatment area: face/scalp or periphery).

The pharmacokinetic set per analyte included all patients who had at least one evaluable predose and postdose pharmacokinetic sample for the respective analyte. A sample was regarded as evaluable if the plasma concentration was above 0 (MUsT‐1) or at or above LLOQ (MUsT‐2). All pharmacokinetic parameters were derived from plasma concentrations of ALA and PpIX using noncompartmental methods. The following parameters were determined: observed maximum baseline‐adjusted plasma concentration (C_max_), t_max_, (area under the baseline‐adjusted plasma concentration‐time curve from time zero to the last sampling time point at which the concentration was at or above LLOQ (AUC_0‐t_), area under the baseline‐adjusted plasma concentration‐time curve, data extrapolated to infinity by calculating AUC = AUC_0‐t_ + C_t_/K_el_, terminal rate constant, assuming first order elimination kinetics (K_el_), and t_1/2_ (calculated by ln2/K_el_). Considerations on reliability of calculated parameters are presented in the supplement.

Statistical analyses were of an exploratory nature without any formal statistical hypotheses. For categorical variables, frequency counts and percentages were used to summarize the results. Descriptive statistics of continuous variables were provided, including number of observations, arithmetic mean, SD, coefficient of variation (if appropriate), and median as well as minimum and maximum. For descriptive statistics of pharmacokinetic parameters and plasma concentrations, the geometric mean, geometric standard deviation, and geometric coefficient of variation were additionally included. For t_max_, only minimum, median, and maximum were provided.

Descriptive statistical analyses were conducted using SAS version 9.2 or higher (SAS Institute, Cary, North Carolina). The noncompartmental pharmacokinetic analysis of the data were accomplished by using Phoenix WinNonlin version 6.0 or higher (Certara, Princeton, New Jersey) (MUsT‐2) and by using SAS version 9.2 or higher (MUsT‐1). Prism version 9.1.0 (GraphPad Software, La Jolla, California) was used for graphical representation of data.

## Results

### Maximal Usage Pharmacokinetic Trial 1

The calibration standards and quality controls of ALA and PpIX sample analysis runs displayed sufficient interbatch precision (<15%) and interbatch accuracy (<±5%) to confirm methodical quality (see Tables S3 and S4). A total of 88.9% of the 36 reanalyzed 5‐ALA samples were within 20% of the mean of the original and repeat result, which meets the recommended requirements.^27^

Altogether, 12 patients were included in the treatment phase of the study and completed the entire course of the study receiving both treatments (placebo [period 1] and BF‐200 ALA [period 2]). Analysis sets are highlighted in Table S4 and demographics and disease parameters are summarized in Table 1A. The safety population consisted of 10 men and 2 women; all patients were White and not Hispanic or Latino. The mean age was 69.8 years. At screening, most of the treatment areas were localized on the forehead. In 1 patient, actinic keratosis lesions were of moderate intensity; in all other patients, actinic keratosis lesions were of mild intensity. The mean total size of the treatment area per patient in both illumination areas (A and B) determined by the investigator on day 1 of both periods was 20.9 cm^2^. Eight of 12 patients received simultaneous illumination with 2 PDT lamps; 4 received illumination with only 1 lamp.

**Table 1:** Patient Demographics, Treatment Parameters, and Disease Characteristics at Baseline

A Patient Demographics, Safety Analysis Set						
			Study			
			MUsT‐1	MUsT‐2		
			Overall (Face) N = 12	Overall N = 32	Face/Scalp N = 16	Periphery N = 16
Sex	n (%)	Female	2 (16.7)	7 (21.9)	1 (6.3)	6 (37.5)
		Male	10 (83.3)	25 (78.1)	15 (93.8)	10 (62.5)
Age, y	Mean ± SD		69.8 ± 5.6	64.3 ± 6.0	64.8 ± 6.0	63.8 ± 6.1
	Range		58‐77	53‐76	56‐73	53‐76
Race	n (%)	White	12 (100)	32 (100)	16 (100)	16 (100)
Ethnicity	n (%)	Not Hispanic or Latino	12 (100)	30 (93.8)	15 (93.8)	15 (93.8)
		Hispanic or Latino	0 (0.0)	2 (6.3)	1 (6.3)	1 (6.3)

B Treatment Parameters and Disease Characteristics at Baseline, Safety Analysis Set						
			Study			
			MUsT‐1	MUsT‐2		
			Overall (Face) N = 12	Overall N = 32	Face/Scalp N = 16	Periphery N = 16
Number of treatment fields	n (%)	1	4 (33.3)	27 (84.4)	14 (87.5)	13 (81.3)
		2	8 (66.7)	5 (15.6)	2 (12.5)	3 (18.8)
Total area of treatment fields	Mean ± SD (cm^2^)		20.9 ± 1.0	60.0 ± 0.0	60.0 ± 0.0	60.0 ± 0.0
Number of lesions within treatment field(s)	Mean ± SD	Diameter ≥ 4 mm	8.3 (2.2)	13.6 (1.8)	13.1 (0.9)	14.1 (2.2)
		Diameter < 4 mm	2.0 (2.0)	2.5 (1.9)	2.3 (1.8)	2.8 (2.0)
Total lesion size^a^	Mean ± SD (cm^2^)		3.2 ± 2.2	4.2 ± 1.4	4.3 ± 1.7	4.1 ± 1.2
Lesion severity^b^	n (%)	Mild/grade 1	ND	105 (24.1)	59 (28.2)	46 (20.4)
		Moderate/grade 2	ND	267 (61.4)	129 (61.7)	138 (61.1)
		Severe/grade 3	ND	63 (14.5)	21 (10.0)	42 (18.6)

MUsT, maximal usage pharmacokinetic trial; N: number of patients; n: number of patients per subgroup; ND, not determined; SD, standard deviation.

^a^The size of each lesion is determined by multiplying largest diameter and perpendicular diameter. For MUsT‐2, the total lesions size sums up the size of all lesions with a diameter ≥4 mm. Lesions with a diameter <4 mm are not included.

^b^Lesion severity was documented for lesions with a diameter ≥4 mm, lesion severity is displayed per lesion; n represents number of lesions per subgroup.

**Figure 2:**
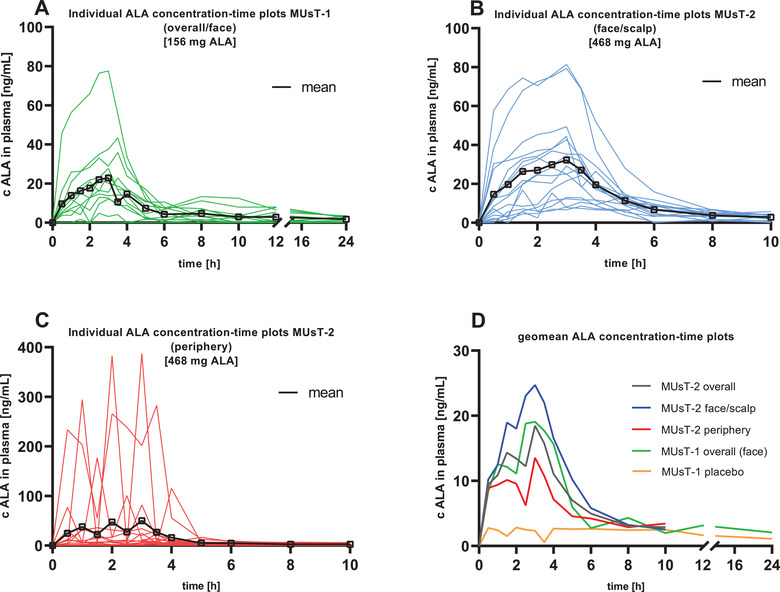
Baseline‐adjusted concentration vs time profiles for ALA. Plasma concentrations of ALA (ng/mL) are plotted against time (h). (A) Individual baseline corrected ALA plasma concentration–time profiles for patients of the pharmacokinetic set of MUsT‐1 (2 g BF‐200 ALA) (n = 12). (B) Individual baseline corrected ALA plasma concentration–time profiles for patients of the pharmacokinetic set of MUsT‐2 (6 g BF‐200 ALA) treated on the face and scalp (n = 16). (C) Individual baseline corrected ALA plasma concentration–time profiles for patients of the pharmacokinetic set of MUsT‐2 (6 g BF‐200 ALA) treated on the body periphery (n = 16). The solid lines in A‐C depict the respective mean concentration versus time profiles for ALA. (D) Geometric mean (geomean) baseline‐adjusted ALA plasma concentrations of the different analysis groups from both trials against time. MUsT‐1 BF‐200 ALA (green, n = 12), MUsT‐1 placebo (yellow, n = 12), MUsT‐2 BF‐200 ALA overall (gray, overall n = 32), MUsT‐2 BF‐200 ALA face/scalp (blue, n = 16), MUsT‐2 BF‐200 ALA periphery (red, n = 16). ALA, 5‐aminolevulinic acid, c, baseline‐adjusted plasma concentration; MUsT, maximal usage pharmacokinetic trial; PDT, photodynamic therapy.

All patients showed ALA concentrations above LLOQ at all sampling time points. In most of the patients, ALA was systemically absorbed, that is, an obvious increase of ALA concentrations was observed after application of 2 g of BF‐200 ALA compared to baseline and the placebo gel (Figure 2A,D). Geometric mean baseline concentrations of ALA were similar across periods. In all patients except for one (see below), baseline concentrations of ALA were similar across periods. Individual plasma concentration‐time profiles of ALA varied strongly between patients after application of the BF‐200 ALA and placebo gel. Maximum geometric mean ALA concentrations were reached at 3 hours after application. Thereafter, ALA was eliminated quickly from plasma returning to approximate baseline levels within 10 hours after application. Baseline‐adjusted total (AUC from time 0 to 24 hours) and C_max_ exposure to ALA were increased after application of the BF‐200 ALA compared to the placebo gel (Figure 2D, Figure S1A). The geometric mean of C_max_ was about 2.5 times of the geometric mean of baseline concentrations. The kinetic parameters which are influenced by the terminal phase, that is, AUC, %AUC from 24 hours to infinity, t_1/2_, and K_el_ could not reliably be determined. These results have thus to be considered with caution. A summary of pharmacokinetic parameters of ALA derived from plasma is given in Table 2 for period 2 (BF‐200 ALA) and in Table S5 for period 1 (placebo).

**Table 2:** Baseline‐Adjusted Pharmacokinetic Parameters for ALA in Plasma (Pharmacokinetic Set)

		MUsT‐1	MUsT‐2		
Pharmacokinetic Parameter		Overall (Face) N = 12	Overall N = 32	Face/Scalp N = 16	Periphery N = 16
AUC_0‐t_ (ng • h/mL)^a^	N Geometric mean Geometric SD/Geometric CV Min‐Max Mean ± SD	11 120.33 1.95/75.0 33.20‐269.44 142.8 ± 75.5	31 89.01 2.7/130.8 13.5‐825.6 146.2 ± 177.6	16 110.03 2.0/75.2 32.7‐353.4 134.3 ± 88.0	15 71.12 3.5/192.8 13.5‐825.6 158.8 ± 242.9
AUC (ng • h/mL)	N Geometric mean Geometric SD/Geometric CV Min‐Max Mean ± SD	10 236.30 1.74/64.64 97.95‐732.02 273.6 ± 176.9	9 138.46 2.0/78.0 35.5‐361.9 166.7 ± 101.2	6 138.41 2.2/90.5 35.5‐361.9 170.5 ± 109.4	3 138.55 1.9/70.8 79.0‐276.9 159.2 ± 104.2
C_max_ (ng/mL)	N Geometric mean Geometric SD/Geometric CV Min‐Max Mean ± SD	11 21.56 2.1/73.63 4.76‐77.53 27.19 ± 20.02	31 27.81 2.9/144.5 6.1‐387.0 53.4 ± 83.6	16 27.93 1.9/72.8 8.6‐81.4 33.9 ± 21.9	15 27.68 4.1/246.9 6.1‐387.0 74.2 ± 116.4
t_max_ (h)	N Median Min‐Max	11 3.0 2.50‐3.50	31 3.02 0.47‐6.0	16 3.0 1.55‐3.5	15 3.42 0.47‐6.0
t_1/2_ (h)	N Geometric mean Geometric SD/Geometric CV Min‐Max Mean ± SD	0	12 2.59 1.9/73.7 1.07‐11.47 3.28 ± 2.90	9 2.86 1.9/74.9 1.52‐11.47 3.64 ± 3.23	3 1.93 1.9/73.3 1.07‐3.92 2.23 ± 1.50

ALA, 5‐aminolevulinic acid; AUC, area under the plasma concentration–time curve; C_max_, observed maximum baseline‐adjusted plasma concentration; CV, coefficient of variation; Max, maximum; Min, minimum; MUsT, maximal usage pharmacokinetic trial; SD, standard deviation; t_1/2_, apparent terminal half‐life (calculated by ln2/K_el_); t_max_, time to reach C_max_.

^a^ t represents the last sampling time point at which the concentration was at or above LLOQ. It is of note that t differs for both studies, with t = 24 hours for MUsT‐1 and t = 10 hours for MUsT‐2.

In 1 patient, baseline concentrations of ALA were about 3‐fold higher in period 2 (BF‐200 ALA) compared to period 1 (placebo gel). A reasonable explanation for this outlier value could not be identified. The outlier value was supposed to be within normal interindividual variability of plasma concentrations.

**Figure 3:**
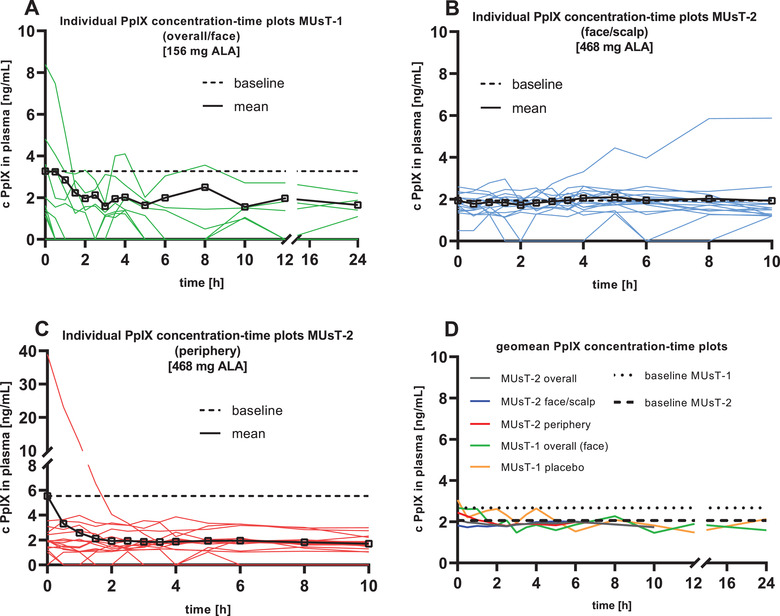
Unadjusted concentration versus time plots for PpIX. Plasma concentrations of PpIX [ng/mL] are plotted against time [h]. As baseline‐adjustment of PpIX generated an abundance of values <0, unadjusted data were chosen for presentation and corresponding mean baseline levels are indicated as horizontal lines. (A) Individual unadjusted PpIX plasma concentration–time profiles for patients of the pharmacokinetic set of MUsT‐1 (2 g BF‐200 ALA) (n = 12). (B) Individual unadjusted PpIX plasma concentration–time profiles for patients of the pharmacokinetic set of MUsT‐2 (6 g BF‐200 ALA) treated on face and scalp (n = 15). (C) Individual unadjusted PpIX plasma concentration–time profiles for patients of the pharmacokinetic set of MUsT‐2 (6 g BF‐200 ALA) (n = 15). The solid lines in A‐C depict the respective mean concentration versus time profiles for PpIX, with all BLQvalues included as 0 in the calculation of the mean. (D) Geometric mean (geomean) unadjusted ALA plasma concentrations of the different analysis groups from both trials against time. MUsT‐1 BF‐200 ALA (green, n = 12), MUsT‐1 placebo (yellow), MUsT‐2 BF‐200 ALA overall (gray, overall n = 30), MUsT‐2 BF‐200 ALA face/scalp (blue, n = 15), MUsT‐2 BF‐200 ALA periphery (red, n = 15). Dashed lines represent the respective overall geometric mean PpIX baseline levels in both trials. ALA, 5‐aminolevulinic acid; BLQ, below limit of quantification; c, unadjusted plasma concentration; MUsT, maximal usage pharmacokinetic trial; PDT, photodynamic therapy; PpIX, protoporphyrin IX.

Concentrations of PpIX were generally low in all patients. Four patients showed concentrations below the LLOQ at all sampling time points (and were thus excluded from the pharmacokinetic set of PpIX (see Table S4). Most of the other 8 patients of the pharmacokinetic set of PpIX showed concentrations below the LLOQ incidentally. Geometric mean baseline concentrations of PpIX were 3.05 ng/mL in period 1 (placebo gel) and 2.67 ng/mL in period 2 (BF‐200 ALA) (Figure 2A). In one patient, baseline concentrations of PpIX were about 2‐fold higher in period 2 compared to period 1 (Figure 3A). A reasonable explanation for this outlier value could not be identified, and a technical error could be excluded. In all other patients, baseline concentrations of PpIX were similar across periods (see Figure 3A, D and Figure S1B).

In none of the patients was an obvious increase of PpIX concentrations observed after application of the BF‐200 ALA gel compared to baseline (Figure 3A), and no obvious difference in concentrations‐time profiles was observed between BF‐200 ALA and placebo gel (Figure 3A and Figure S1B). After application of the BF‐200 ALA and placebo, a slight decrease of geometric mean plasma concentrations of PpIX compared to baseline was observed, and individual plasma concentration–time profiles of PpIX varied strongly between patients after application of the BF‐200 ALA and placebo gel (Figure 3A and Figure S1B). Due to the high number of PpIX concentrations below LLOQ and 0 concentrations after baseline adjustment, Figure 3A and D show unadjusted concentration‐time profiles for PpIX. Baseline‐adjusted pharmacokinetic parameters could only be calculated in 1 patient after application of the BF‐200 ALA (Table 3). Descriptive statistics of baseline‐adjusted pharmacokinetic parameters C_max_ and AUC from time 0 to 24 hours of PpIX were still provided for all 8 patients included in the PpIX pharmacokinetic set (Table 3 for period 2 [BF‐200 ALA] and Table S5 for period 1 [placebo]). In summary, in none of the patients, was an obvious difference of PpIX concentrations observed after application of BF‐200 ALA compared to baseline and placebo, that is, metabolism of ALA to PpIX was not increased under maximal use conditions.

**Table 3:** Baseline‐Adjusted Pharmacokinetic Parameters for PpIX in Plasma (Pharmacokinetic Set)

		MUsT‐1	MUsT‐2		
PK Parameter		Overall (Face) N = 8	Overall N = 30	Face/Scalp N = 15	Periphery N = 15
AUC_0‐t_ (h • ng/mL)^a^	N Geo. Mean Geo SD/Geo CV Min‐Max Mean ± SD	1 0.07 N.C./N.C. 0.07–0.07 0.07 ± N.C.	22 0.8 4.4/281.3 0.06–13.48 1.9 ± 2.9	13 0.88 4.6/307.2 0.06–13.48 2.2 ± 3.5	9 0.68 4.4/280.2 0.06–5.07 1.4 ± 1.6
C_max_ (ng/mL)	N Geo. Mean Geo SD/Geo CV Min‐Max Mean ± SD	1 0.29 N.C. 0.29–0.29 0.29 ± N.C.	22 0.42 2.2/90.8 0.12–3.17 0.58 ± 0.64	13 0.48 2.2/91.7 0.12–3.17 0.67 ± 0.78	9 0.34 2.2/90.0 0.12–1.22 0.45 ± 0.36
t_max_ (h)	N Median Min‐Max	1 0.5 0.5–0.5	22 3.76 0.47–9.88	13 4.0 1.00–9.88	9 3.52 0.47–8.0

AUC, area under the plasma concentration time curve; C_max_, observed maximum baseline‐adjusted plasma concentration; Geo, geometric; CV, coefficient of variation; Max, maximum; Min, minimum; MUsT, maximal usage pharmacokinetic trial; N, number of evaluable patients; n, number of non‐missing values; N.C., not calculated; PK, pharmacokinetic; PpIX, protoporphyrin IX; SD, standard deviation.

^a^ t represents last sampling time point at which the concentration was at or above the lower limit of quantification. It is of note that t differs for both studies, with t = 24 hours for MUsT‐1 and t = 10 hours for MUsT‐2.

Local tolerability was assessed 24 hours after drug application in period 1 and 24 hours after drug application and at the follow‐up visit 7 days after period 2. The frequency of events reported for period 2 (BF‐200 ALA) is aggregated over these 2 time points. An overview of application site skin reactions and discomfort is given in Table 4A. Skin reactions occurred more often after application of BF‐200 ALA compared to placebo. After application of placebo and subsequent illumination, mild dryness occurred in 8 patients, and mild itching occurred in 1 patient. After application of BF‐200 ALA and subsequent illumination, all patients showed skin reactions from the predefined categories (erythema, dryness, burning, erosion, edema, and itching). The most frequently observed skin reaction was erythema followed by edema, dryness, erosion, burning, and itching. The skin reactions were mostly of mild or moderate intensity, apart from 1 patient, who showed severe erythema and erosion. Immediately after illumination following placebo application, none of the patients developed pain in the treatment area. After illumination following treatment with BF‐200 ALA, all patients developed pain, mostly of moderate intensity. None of the patients received 1 g of acetaminophen as preventive measure for pain before the illumination in period 1 (placebo), while 10 patients received acetaminophen before illumination in period 2 (BF‐200 ALA). Apart from the application site skin reactions and pain at the application site, 9 at least possibly related TEAEs were observed in 4 of the 12 patients: 6 local TEAEs were observed in 2 patients (eyelid edema, swelling of the face, application site erosion, application site pruritus, and application site pain in 1 patient, and feeling hot in another patient) and 3 events of headache were observed in 3 patients. No deaths and no serious AEs occurred. No discontinuations due to an AE were reported. All TEAEs had recovered by the end of the study.

**Table 4:** Safety Assessment and Pain During PDT

A Application Site Skin Reactions and Discomfort in MUsT‐1		
	Placebo N = 12	BF‐200 ALA^a^ N = 12
	n (%)	n (%)
Burning	0 (0)	7 (50.0)
Dryness	8 (66.7)	10 (33.3)
Edema	0 (0)	11 (91.7)
Erosion	0 (0)	9 (16.7)
Erythema	0 (0)	12 (100)
Itching	1 (8.3)	7 (16.7)
Pain	0 (0)	12 (100)

B Application Site Skin Reactions and Discomfort in MUsT‐2			
	Overall^b^ N = 32	Face/Scalp^b^ N = 16	Periphery^b^ N = 16
	n (%)	n (%)	n (%)
Discoloration	2 (6.3)	1 (6.3)	1 (6.3)
Erosion	2 (6.3)	1 (6.3)	1 (6.3)
Erythema	32 (100)	16 (100)	16 (100)
Exfoliation	13 (40.6)	10 (62.5)	3 (18.8)
Fissure	2 (6.3)	1 (6.3)	1 (6.3)
Hyperesthesia	3 (9.4)	2 (12.5)	1 (6.3)
Induration	4 (12.5)	2 (12.5)	2 (12.5)
Edema	25 (78.1)	16 (100)	9 (56.3)
Pain	32 (100)	16 (100)	16 (100)
Paresthesia	5 (15.6)	4 (25.0)	1 (6.3)
Pruritus	10 (31.3)	4 (25.0)	6 (37.5)
Scab	11 (34.4)	5 (31.3)	6 (37.5)
Vesicles	3 (9.4)	1 (6.3)	2 (12.5)
Warmth	5 (15.6)	4 (25.0)	1 (6.3)

C Pain on 11‐Point Numeric Rating Scale During PDT in MUsT‐2			
	Overall N = 32	Face/Scalp N = 16	Periphery N = 16
n (%)	32 (100)	16 (100)	16 (100)
Mean ± SD	7.1 ± 2.3	8.1 ± 2.0	6.0 ± 2.1
Median	8.0	9.0	6.0
Range	2‐10	4‐10	2‐9

ALA, aminolevulinic acid; MUsT, maximal usage pharmacokinetic trial; PDT, photodynamic therapy; SD, standard deviation.

^a^Number of patients with application site reactions after PDT with BF‐200 ALA aggregated over the 7‐day follow‐up.

^b^ Number of patients with application site reactions after PDT aggregated over the 28‐day follow‐up.

### Maximal Usage Pharmacokinetic Trial 2

The calibration standards and quality controls of ALA and PpIX sample analysis runs displayed sufficient interbatch precision (<15%) and interbatch accuracy (<±5%) to confirm methodical quality (see Tables S3 and S4). A total of 70.8% of the 48 reanalyzed PpIX samples and 80.6% of the 72 reanalyzed ALA samples were within 20% of the mean of the original and repeat result, that is, the incurred sample reanalysis guideline requirements were met.

In total, 46 patients were enrolled, and a total of 32 patients were included in the treatment phase of the study. Of these, 16 patients were treated on the face or scalp and 16 on the body periphery. For a tabular view of patient disposition, see Table S4. Demographic data are summarized in Table 1A. All 32 patients were White, and 2 patients were Hispanic or Latino. The mean age was 64.3 years. The mean total area of treatment fields included in the study was 60.0 cm^2^. The number and size of the lesions within treatment field(s) and the severity were comparable for both strata, thus allowing the comparison of pharmacokinetic and safety data. A summary of treatment field and AK target lesion assessments is presented overall and by strata in Table 1B.

With one exception, all patients showed ALA concentrations above the LLOQ at all sampling time points. Hence, ALA was systemically absorbed; that is, a measurable increase of ALA concentrations was observed after application of BF‐200 ALA (see Figure 2B‐D). Maximum overall geometric mean ALA concentrations were reached at 3 hours after application. Thereafter, ALA was eliminated quickly from plasma, returning to approximate baseline levels within 10 hours after application. Individual plasma concentration‐time profiles of ALA varied strongly between patients after application of BF‐200 ALA (Figure 2B,C). Variability in baseline‐adjusted plasma concentrations was higher in the stratum periphery (Figure 2C) than in the stratum face/scalp (Figure 2B), which was mainly driven by the high fluctuating profiles of 2patients.

Overall, there was only little systemic absorption of the total ALA dose; the overall geometric mean of C_max_ was about 3.3 times the overall geometric mean of baseline concentration. Moreover, total, but not maximal, exposure was slightly higher in the stratum face/scalp than in the stratum periphery. Median overall t_max_ was observed at 3.0 hours after application of BF‐200 ALA. Less than 40% of the patients had reliable data for the evaluation of K_el_, t_1/2_, and AUC. Therefore, the results of these parameters should be considered with caution.

For PpIX, a high number of data points ≤0 occurred after baseline adjustment. Hence, the unadjusted concentration‐time profiles are presented in Figure 3B through D. No measurable increase of PpIX concentrations was observed after application of BF‐200 ALA compared to baseline. Unadjusted overall geometric mean plasma concentrations of PpIX after application of the drug rather oscillated in a range of up to approximately 5 ng/mL (Figure 3B,C). No differences between unadjusted geometric mean PpIX plasma concentration–time curves were found between both strata (Figure 3B‐D). Baseline‐adjusted total (AUC from time 0 to 10 hours) and C_max_ exposure to PpIX was increased minimally in 22 of 30 patients in the PpIX pharmacokinetic set after application of the BF‐200 ALA (see Table 3).

The mean baseline PpIX plasma concentration in one patient (51.1 ng/mL) was clearly higher than the normal physiological range in plasma of healthy adults. This is reported in the literature to be below 10 ng/mL.^28^ This patient had also shown a very high baseline for ALA (41.9 ng/mL). It is of note that both PpIX and ALA plasma concentrations in this patient decreased after dosing, and thus the lowest PpIX plasma concentration (1.03 ng/mL) was measured 10 hours after administration of BF‐200 ALA. In another patient, the unadjusted PpIX plasma concentration–time curve showed a delayed slight increase in the PpIX plasma concentration. The highest PpIX plasma concentration (5.87 ng/mL) was detected 10 hours after application of BF‐200 ALA compared to the individual baseline of 2.7 ng/mL. Since the ALA plasma concentration in the patient returned to the endogenous level (see Figure 2B), it was inferred that the PpIX plasma concentration would also return to baseline with delay. The only clinically significant safety finding was a significant number of bacteria and an increased white blood cell count in urine at the time of dosing, indicating that this patient was likely suffering from a urinary tract infection. Given that no signs of photosensitivity could be derived from the TEAE profile reported for the patient, and in conjunction with the full clearance of ALA from plasma after 8 hours, there is no indication that the observed time‐delayed increase in the PpIX plasma concentration was associated with a risk for the patient's safety.

**Figure 4:**
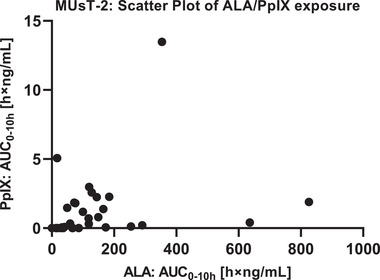
Scatter plot of AUC_0‐10h_ for ALA and PpIX in MUsT‐2. Individual AUC_0‐10h_ were derived from baseline‐adjusted plasma concentrations of ALA and PpIX: Data pairs were plotted to analyze for a relationship between exposure to parent drug (ALA) and its photosensitizing key metabolite (PpIX) in plasma. ALA plasma exposure from BF‐200 ALA appears to be no driver of PpIX in plasma in this study. ALA, 5‐aminolevulinic acid; AUC_0‐10h_, area under the plasma concentration–time curve from time 0 to 10 hours; MUsT, maximal usage pharmacokinetic trial; PpIX, protoporphyrin IX

As an additional analysis, patients’ exposure in plasma to parent drug ALA and metabolite PpIX were compared by plotting the baseline‐adjusted AUC from time 0 to 10 hours (AUC_0‐10h_) of ALA (*x* axis) to the baseline‐adjusted AUC_0‐10h_ of PpIX (*y* axis) (Figure 4). Based on this analysis, there seems to be no relationship between AUC_0‐10h_ of ALA and AUC_0‐10h_ of PpIX. Hence, ALA plasma exposure from BF‐200 ALA seems not to be a significant driver of PpIX in plasma in this study.

The overall incidence of patients with treatment‐emergent application site conditions was 100% for patients treated on the face/scalp and for patients treated in the periphery. In both strata, the most commonly reported TEAEs were those of the application site. Application site erythema and application site pain were the most common individual events in both strata (see Table 4B). The most commonly reported severe reaction was application site pain. The majority of severe events resolved within 1 to 2 days and most events experienced overall were reported as resolved by the end of the study. Mean pain intensity reported during PDT was measured on an 11‐point numeric rating scale and is summarized in Table 4C. The mean of the pain intensities reported by patients treated on the face/scalp was numerically higher than the mean reported by patients treated in the periphery. There were no deaths, SAEs, and other clinically meaningful AEs or clinically relevant findings in the safety laboratory analyses related to the study treatment during and until the end of the study.

## Discussion

The primary objective of both MUsTs was to assess the pharmacokinetics of the parent drug 5‐ALA and its active metabolite PpIX in the systemic circulation after a single, field‐directed PDT applying either 2 g (MUsT‐1) or 6 g (MUsT‐2) of BF‐200 ALA 10% gel in conjunction with red‐light PDT under maximal use conditions in patients with AK.

In MUsT‐1, in which 1 tube of BF‐200 ALA was applied on treatment fields of 20 cm^2^ located on the face or forehead (total dose, 156 mg ALA), no systemic elevation of PpIX levels was observed. A slight and transient elevation of systemic ALA levels was detected, which was well below the daily rate of ALA synthesis (200‐354 mg).^29^ ALA was quickly eliminated from plasma and returned to baseline concentrations within 10 hours after BF‐200 ALA administration. Further, the analysis of the placebo period revealed no circadian changes in endogenous ALA or PpIX, such that omission of the placebo period and shortening of the sampling period to 10 hours after application of BF‐200 ALA seemed scientifically justified in MUsT‐2.

MUsT‐2 was designed to assess pharmacokinetics and safety of PDT treatment in 2 strata (face/scalp vs periphery [neck/trunk/extremities]) to account for a possible influence of varying epidermal thickness between different body regions^30^, ^31^ on the systemic absorption of study medication as well as tolerability of treatment in an expanded treatment field of 60 cm^2^.

The pharmacokinetic results of MUsT‐2 demonstrated a transient increase of systemic total and maximum exposure to ALA, which was slightly higher in the stratum face/scalp than in the stratum periphery. Possible explanations might be a generally higher skin temperature of face and scalp in comparison to peripheral skin,^32^ as ALA penetration into the skin is known to be a temperature‐dependent process,^33^, ^34^ and additionally less epidermal thickness, especially in facial skin.^30^, ^31^

The overall geometric mean of C_max_ determined for ALA was about 3.3 times the overall geometric mean of the baseline concentrations, indicating that only a minor fraction of the topical dose is systemically absorbed: From a total topical dose of 468 mg, the overall geometric mean baseline‐adjusted AUC indicates that the predominant portion of the topical ALA dose remains locally in the skin and that relative topical bioavailability is very low. ALA was eliminated quickly from plasma returning to approximate baseline levels within 10 hours. The results obtained in MUsT‐2 are similar to the results of MUsT‐1, although a 3‐fold higher dose was administered to a 3‐fold larger area. The overall baseline‐adjusted geometric mean AUC was even higher in MUsT‐1 compared to the overall geometric mean AUC in MUsT‐2. This difference may be related to the different treatment areas, as facial skin is characterized by less epidermal thickness compared to other body parts, particularly scalp and hands.^30^, ^31^ Pharmacokinetic results further demonstrated that systemic PpIX exposure after administration of both ALA doses was similarly minimal.

Taking into consideration the available data from the scientific literature, the observed elevation in the plasma concentration of ALA in both studies is not expected to yield any noticeable clinical effect and thus regarded as uncritical. PpIX should be regarded as the key factor for systemic safety. Mild systemic adverse effects such as nausea and vomiting have been reported in patients with PpIX plasma concentrations of 0.56 to 1.59 μg/mL following oral dosing with 30 to 60 mg/kg ALA.^35^ In both MUsTs, PpIX plasma concentrations were almost not measurably increased after BF‐200 ALA application, which correlates with the fact that, except for 4 patients with headache, no systemic TEAE was observed. And as the extent of ALA exposure does not seem to be predictive for the extent of PpIX exposure in plasma (Figure 4), the safety risk from systemic exposure is equally minimal after receiving a dose of either 1 or 3 tubes of BF‐200 ALA.

All patients in both studies had at least 1 application site skin reaction or discomfort after PDT with BF‐200 ALA. In MUsT‐2, the overall incidence of related treatment‐emergent application site conditions was comparable for both strata, with certain exceptions as highlighted in the results section. In both studies, application site pain, erythema, and edema were the most frequently recorded events in connection to BF‐200 ALA (Tables 4A and B). This aligns with previous results with patients treated with BF‐200 ALA for AK^15^, ^16^, ^17^, ^18^ and is to be expected as the therapeutic principle of PDT is based on phototoxic effects of PpIX, which is locally synthesized from ALA in the diseased skin.^36^ In MUsT‐2, pain was more specifically analyzed using an 11‐point numeric rating scale. Patients treated on the face/scalp reported numerically higher pain intensities than patients treated in the periphery. Physiological differences between strata in terms of density of nerve endings^37^ and skin temperature^32^ might also have contributed to the observed numerical differences in mean pain intensity. In comparison to previous studies, the mean pain intensity assessed in MUsT‐2 was numerically slightly higher.^16^, ^17^, ^18^ When comparing pain intensities across both studies, it is noteworthy that in MUsT‐1 most patients received acetaminophen ≈1 hour before illumination as a pain‐preventive measure, while this was not the case in MUsT‐2. Given that PDT pain was already described to be related to the size of treatment fields,^38^ a higher pain intensity during treatment of expanded treatment fields was to be expected. However, application site pain decreased shortly after illumination and was well manageable, as all illuminations were performed without interruption. Finally, it is of note that tolerability was not affected by using >1 lamp simultaneously for illumination.

## Conclusions

Evaluation of pharmacokinetics indicates that the systemic exposure profile after treatment with 3 tubes of BF‐200 ALA is not significantly different from the systemic exposure profile after treatment with 1 tube of BF‐200 ALA. Based on the obtained safety as well as pharmacokinetics data, no safety concern due to high PpIX exposure is apparent upon application of 2 to 6 g of BF‐200 ALA for PDT of extensive AK in treatment fields located on the face/scalp or periphery. Evidently, systemic metabolism of ALA to PpIX is not increased under maximal use conditions. In summary, the studies demonstrate that field‐directed treatment of AK with a dose of up to 6 g BF‐200 ALA is safe on surface areas of up to 60 cm^2^.

## Conflicts of Interest

B.N., K.O., B.S., C.Z., and H.L. are or were employed by the sponsoring company, and B.N., K.O., B.S., and H.L. hold either stock or stock options of Biofrontera AG. The remaining authors declare no conflicts of interest.

## Author Contributions

B.S. and B.N. provided clinical pharmacology expertise, designed the studies, and analyzed the data. J.D. and T. Papusha provided medical expertise, advised on study design, and collected the data. O.C. and T. Philippi provided bioanalytical expertise, developed and validated the assays, and collected the data. S.H. performed the pharmacokinetics and statistical analysis of MUsT‐2. C.Z. contributed to study design of MUsT‐2 and provided safety expertise and editorial assistance. B.N., K.O., and O.C. wrote the manuscript. H.L. provided pharmacological guidance, advised on study design, and revised the manuscript for important intellectual content. All authors critically revised and approved the final manuscript.

## Funding Information

These clinical studies were sponsored and funded by Biofrontera Bioscience GmbH.

## Data Sharing Statement

Data supporting the results of this publication are publicly available in the ClinicalTrials.gov database under http://www.clinicaltrials.gov/ct2/show/NCT04319159

## Supporting information

SUPPLEMENTARY INFORMATION
