# Supplementary material for: Clinical Pharmacokinetics and Safety of a 10% Aminolevulinic Acid Hydrochloride Nanoemulsion Gel (BF‐200 ALA) in Photodynamic Therapy of Patients Extensively Affected With Actinic Keratosis: Results of 2 Maximal Usage Pharmacokinetic Trials
Source: Clin Pharmacol Drug Dev. 2021 Oct 11;11(4):535–50. doi: 10.1002/cpdd.1023 (PMC9293336; doi:10.1002/cpdd.1023)

**Novak *et al.*,** Clinical Pharmacokinetics and Safety of a 10% aminolevulinic acid hydrochloride nanoemulsion gel (BF-200 ALA) in Photodynamic Therapy of patients extensively affected with Actinic Keratosis: results of two maximal usage pharmacokinetic trials (MUsT)

**Supplementary Material:**

**Supplementary Methods:**

MUsT-2: Accuracy and Precision from Method Validation

ALA: During validation, the method displayed inter batch precision (%) of 7.8, 6.6, 5.1, 6.6, and 7.9, and intra-batch precision (%) of 3.4 to 9.2, 1.5 to 3.2, 1.4 to 7.7, 0.7 to 2.8, and 1.0 to 3.5 at the LLOQ, low-, bio-, med-, and high-QC levels, respectively. The inter-batch accuracy (%) of the method was 108.1, 105.3, 99.3, 98.8, and 106.1 and the intra-batch accuracy (%) of the method was 99.5 to 115.0, 97.8 to 113.2, 95.6 to 100.9, 89.9 to 105.2, and 92.8 to 112.3 at the LLOQ, low-, bio-, med-, and high-QC, respectively.

PpIX: During validation, the method displayed inter batch precision (%) of 7.4, 10.3, 8.0, and 5.7 and intra-batch precision (%) of 2.9 to 6.9, 3.4 to 7.3, 3.3 to 11.0, and 4.4 to 7.8 at the LLOQ, low-, med-, and high-QC levels, respectively. The inter-batch accuracy (%) of the method was 105.2, 98.8, 101.0, and 106.8 and the intra-batch accuracy (%) of the method was 98.5 to 111.4, 93.1 to 103.1, 98.5 to 103.9, and 105.9 to 107.5 at the LLOQ, low-, med-, and high-QC levels, respectively.

Reliability of pharmacokinetic parameters

AUC_0-t_ and AUC were regarded as unreliable if more than three consecutive results were missing or if the concentrations were quantifiable for fewer than five time points. C_max_ and t_max_ were regarded as unreliable if the maximum was observed preceding or following a sample with missing data. In case of multiple peaks, C_max_ and t_max_ were to be referred to the highest measured concentration even if there were earlier peaks. In case of two or more samples with the same concentration, t_max_ was to be referred to the earlier of these.

The calculation of K_el_ was considered unreliable in case of r²<0.8. K_el_, t_1/2_, AUC were only determined in patients in whom the log-linear terminal phase could clearly be defined. The value of AUC was considered unreliable but was reported if the terminal area beyond the last quantified sample was greater than 20% of the total AUC. If any pharmacokinetic parameter was classified as unreliable, all calculations that used this parameter were considered missing.

**Supplementary Tables:**

Table S1: Instrumental conditions of bioanalysis MUsT-1

| **5-aminolevulinic acid** | |  |  | **Protoporphyrin IX** | |  |  |  |
| --- | --- | --- | --- | --- | --- | --- | --- | --- |
| **HPLC: FLUX™ Rheos 2200, Flom 502 column oven and CTC HTC PAL auto-sampler** | | | | **HPLC: Waters™ P14 Pump, AS18 Auto sampler and SO18 Column oven** | | | | |
| Column: | Phenomenex Synergi 2.5u Hydro-RP: 2.5 μm, 100 x 2 mm | | | Column: | ACQUITY® UPLC BEH C18 | | | |
| Column oven: | 55°C | | |  | Column, 130Å, 1.7 μm, 2.1 mm x | | | |
| Mobile Phase A: | 0.6% (w/v) Ammonium formate in 0.5% aqueous formic acid | | |  | 50mm | | | |
| Mobile Phase B: | 0.5% (v/v) formic acid in ACN | | | Column oven: | 50°C | | | |
| Auto-sampler temperature | 4°C | | | Mobile Phase A: | 0.1% formic acid in H_2_O | | | |
| Flow rate: | 0.35 mL/min | | | Mobile Phase B: | 0.1% formic acid in ACN | | | |
| Gradient: | Time (min) | %B | | Auto-sampler temperature | 5°C | | | |
|  | 0.0 | 0.0 | | Flow rate: | 0.50 – 0.75 mL/min | | | |
|  | 1.0 | 0.0 | | Gradient: | Time (min) and Flow (mL/min) | | | %B |
|  | 4.5 | 50.0 | |  | 0.0 and 0.5 | | | 20.0 |
|  | 4.8 | 50.0 | |  | 0.5 and 0.5 | | | 20.0 |
|  | 4.9 | 95.0 | |  | 1.5 and 0.5 | | | 98.0 |
|  | 6.0 | 95.0 | |  | 1.7 and 0.75 | | | 98.0 |
|  | 6.1 | 0.0 | |  | 2.7 and 0.75 | | | 98.0 |
|  | 7.0 | 0.0 | |  | 3.0 and 0.5 | | | 98.0 |
| Injection volume: | 10 µL | | |  | 3.5 and 0.5 | | | 20.0 |
| Auto-sampler Rinse | Rinse 1 and 2: 1% formic acid in (1:1, v/v) MeOH: ACN | | |  | 4.5 and 0.5 | | | 20.0 |
| Auto-sampler conditions | Pre Clean with Solvent 1 and 2, and Pre Clean with Sample | | 0 | Injection volume: | 10 µL | | | |
|  | Syringe | | 25 μL | Auto-sampler Rinse | ACN:MeOH:H_2_O:DMSO:formic acid (80:80:20:20:2 v/v/v/v/v) | | | |
|  | Filling Speed and Injection Speed | | 5 μL/s | Auto-sampler Conditions | Purge solution | | 90:10 H_2_O:ACN (v/v) | |
|  | Post Clean with Rinse 1 and 2 | | 5 |  | Syringe | | 10 μL | |
|  | Valve Clean with Solvent 1 and 2 | | 2 |  | Pre Clean with rinse solution | | 5 Sec | |
| **Mass Spectrometer : SCIEX™ Triple Quad 5000** | | | |  | Post Clean with rinse solution | | 10 Sec | |
|  | | | |  | Injection Speed | | 10 μL/s | |
| **uad6500+** | | | | **Mass Spectrometer : Waters™ XEVO TQ-S** | | | | |
| Parameter | 5-Aminolevulinic acid | | L-Lysine-^13^C_6_,^15^N_2_ hydro chloride | Parameter | Protoporphyrin IX | | Midazolam-d_4_ | |
| Interface | ESI, +ve ION | | | Interface | ESI, +ve ION | | | |
| CAD | 8 | | | Desolvation temperature | 500 | | | |
| CUR | 25 | | | [°C] |  |  |  |  |
| GS1 | 65 | | | Desolvation gas flow | 1000 | | | |
| GS2 | 35 | | | Source temperature [°C] | 150 °C | | | |
| TEM | 550°C | | | Capillary (kV) | 3.50 | | | |
| DP | 150 | | 120 | Cone [V] | 40 | | | |
| EP | 10 | | |  |  |  |  |  |
| CE | 25 | | 21 | Cone gas flow [L/h] | 150 | | | |
| CXP | 20 | | | CE | 48 | 30 | | |
| MRM (m/z) | 188.1→114.2 | | 211..2→90.0 | Multiplier [V] | 650 | | | |
| Dwell time (msec) | 75 | | | MRM (m/z) | 562.70 → 504.85 | | 330.20→ 295.00 | |
|  |  |  |  |  |  |  |  |  |

CAD: collision activated dissociation gas ; CE: collision energy; CUR: curtain gas ; CXP: collision cell exit potential ; DP: declustering potential ; EP: entrance potential ; GS1: ion source gas 1 ; GS2: ion source gas 2 ; TEM: is temperature ; MRM (m/z) multiple reaction monitoring (mass/charge)

Table S2: Instrumental conditions of bioanalysis MUsT-2

| **5-aminolevulinic acid** | |  |  | **Protoporphyrin IX** | |  |  |
| --- | --- | --- | --- | --- | --- | --- | --- |
| **UHPLC: SCIEX™ ExionLC** | | | | **UHPLC: SCIEX™ ExionLC** | | | |
| Column: | ACE UltraCore 2.5 | | | Column: | ACE 3 AQ, C_18_ 50 x 3.0 mm | | |
|  | SuperPhenylHexyl 50 x 2.1mm | | | Column oven: | 50°C | | |
| Column oven: | 55°C | | | Mobile Phase A: | H_2_O:ACN 90:10 v/v with 0.1% formic acid | | |
| Mobile Phase A: | 0.126% (w/v) Ammonium formate in 0.5% aqueous Formic Acid | | | Mobile Phase B: | ACN in 0.1% formic acid | | |
| Mobile Phase B: | 0.5% (v/v) Formic Acid in Acetonitrile | | | Auto-sampler temperature | 5°C | | |
| Auto-sampler temperature | 5°C | | | Flow rate: | 0.50 mL/min | | |
| Flow rate: | 0.35 mL/min | | | Gradient: | Time | %B | |
| Gradient: | Time (min) | %B | |  | 0.00 | 50 | |
|  | 1.00 | 0.0 | |  | 0.50 | 50 | |
|  | 5.00 | 12.5 | |  | 0.75 | 80 | |
|  | 5.25 | 50.0 | |  | 3.50 | 100 | |
|  | 5.50 | 95.0 | |  | 5.00 | 100 | |
|  | 7.00 | 95.0 | |  | 5.10 | 50 | |
|  | 7.10 | 0.0 | |  | 7.50 | 50 | |
|  | 9.00 | 0.0 | | Injection volume: | 2 µL | | |
| Injection volume: | 5 µL | | | Auto-sampler Rinse | ACN:MeOH:Formic Acid (50:50:0.3% v/v/v) | | |
| Auto-sampler Rinse | Deionised Water | | | Integrated Valco Valve | Time (minutes) | | Position |
| Integrated Valco Valve | Time (minutes) | | Position |  | 0.0 | | to waste |
|  | 0.0 | | to waste |  | 1.50 | | to MS detector |
|  | 4.0 | | to MS detector |  | 3.20 | | to waste |
|  | 6.0 | | to waste | **Mass Spectrometer : SCIEX™ Triple Quad 6500+** | | | |
| **Mass Spectrometer : SCIEX™ Triple Quad 6500+** | | | |  |  |  |  |
|  | | | | Parameter | Protoporphyrin IX | | Protoporphyrin IX D6 |
|  |  |  |  | Interface | ESI Turbo Spray IonDrive, +ve ION | | |
| Parameter | 5-Aminolevulinic acid | | 5-Aminolevulinic acid (^13^C_2_, ^15^N) | CAD | 10 | | |
| Interface | APCI IonDrive™ Turbo V Source, +ve | | | CUR | 20 | | |
| CAD | 8 | | | GS1 | 80 | | |
| CUR | 20 | | | GS1 | 80 | | |
| GS1 | 40 | | | TEM | 550°C | | |
| TEM | 250°C | | | ISV | 5500 | | |
| DP | 41 | | | DP | 55 | | |
| EP | 10 | | |  |  |  |  |
| CE | 17 | | | EP | 11 | | |
| CXP | 22 | | | CE | 55 | | |
| MRM (m/z) | 188.1→114.0 | | 191.1→117.0 | CXP | 12 | | |
| Dwell time (msec) | 150 | | | MRM (m/z) | 563.3→504.3 | | 569.3→510.3 |
|  |  |  |  | Dwell time (msec) | 150 | | |
|  |  |  |  |  |  |  |  |

CAD: collision activated dissociation gas ; CE: collision energy; CUR: curtain gas ; CXP: collision cell exit potential ; DP: declustering potential ; EP: entrance potential ; GS1: ion source gas 1 ; TEM: is temperature ; MRM (m/z) multiple reaction monitoring (mass/charge)

Table S3: Performance of bioanalytical methods

| **Parameter** | **ALA** | **PpIX** |
| --- | --- | --- |
| MUsT-1 | | |
| Lower level of quantification (LLOQ, [ng/mL]) | 1.000 | 1.000 |
| Inter-batch precision of calibration standards (%) | 1.8-7.4 | 3.8-8.4 |
| Inter-batch accuracy of calibration standards (%) | 97.9-100.8 | 93.5-96.0 |
| *Inter-batch precision of quality control samples (%) | 5.1-12.4 | 9.1-10.9 |
| **Inter-batch accuracy of quality control samples (%) | 92.1-101.7 | 91.0-103.0 |
| MUsT-2 | | |
| Lower level of quantification (LLOQ, [ng/mL]) | 1.000 | 1.000 |
| Inter-batch precision of calibration standards (%) | 1.9-4.8 | 4.7-7.7 |
| Inter-batch accuracy of calibration standards (%) | 97.2-103.0 | 94.6-103.0 |
| *Inter-batch precision of quality control samples (%) | 3.1-5.2 | 8.8-10.3 |
| **Inter-batch accuracy of quality control samples (%) | 99.7-101.8 | 101.1-102.6 |

*Precision calculated for mid- and high-QC samples includes the additional variability of the endogenous concentration between the matrices used. Low-QC was prepared in surrogate matrix.

**Accuracy for med- and high-QC samples calculated as the average of individual accuracy values in every single batch based on the calculated endogenous concentration.

ALA: 5-aminolevulic acid, LLOQ: lower limit of quantification, MUsT: maximal usage pharmacokinetic trial, PpIX: protoporphyrin IX

Table S4: Patient disposition and data analysis sets

| **Set** | **Patients, n (%)** |
| --- | --- |
| MUsT-1 (ALA-AK-CT006) | |
| Enrolled set (ENR) | 13 (100) |
| Safety Analysis Set (SAF) | 12 (92.3) |
| Treated set (TS) | 12 (92.3) |
| ALA Pharmacokinetic set (ALA PKS) | 12 (92.3) |
| PpIX Pharmacokinetic set (PpIX PKS) | 8 (61.5) |
| MUsT-2 (ALA-AK-CT015) | |
| Enrolled set 2* (ENR2) | 46 (100) |
| Safety Analysis Set (SAF) | 32 (69.6) |
| Treated set (TS) | 32 (69.6) |
| ALA Pharmacokinetic set (ALA PKS) | 32 (69.6) |
| PpIX Pharmacokinetic set (PpIX PKS) | 30 (65.2) |

* includes all patients who provided informed consent once (two patients were re-screened due to COVID-19 implications; these patients were included with their 2^nd^ screening ID only)

ALA: 5-aminolevulic acid, n: number of patients in set, PpIX: protoporphyrin IX

Table S5: Pharmacokinetic parameters after Placebo (period 1) in MUsT-1

|  |  | **ALA** | **PpIX** |
| --- | --- | --- | --- |
| **PK Parameter** |  | **Overall (Face)**  **N = 12** | **Overall (Face)l**  **N = 8** |
| AUC_0-24 h_  [h×ng/mL] | n  Geo. Mean  Geo SD / Geo CV  Min. – Max.  Mean ± SD | 12  22.36  3.28 / 176.07  1.40 – 85.23  34.96 ± 27.06 | 2  1.15  4.27 / 268.79  0.41 – 3.22  1.82 ± 1.99 |
| C_max_  [ng/mL] | n  Geo. Mean  Geo SD/Geo CV  Min. – Max.  Mean ± SD | 12  4.47  1.56 / 46.76  1.40 – 6.94  4.81 ± 1.61 | 2  1.23  3.11 / 161.96  0.55 – 2.75  1.65 ± 1.55 |
| t_max_ [h] | n  Median  Min. – Max. | 12  7.00  1.00 – 10.03 | 2  1.50  1.50 – 1.50 |

AUC: area under the plasma concentration time curve, C_max_: observed maximum baseline-adjusted plasma concentration, Geo: geometric, CV: coefficient of variation, Max: maximum, Min: minimum, MUsT: maximal usage pharmacokinetic trial, N: number of evaluable patients, n: number of non-missing values, PK: pharmacokinetic, PpIX: protoporphyrin IX, SD: standard deviation, t_max_: time to reach C_max_.

Legends to Supplementary Figures:

**Figure S1:** Baseline-adjusted (ALA) and unadjusted (PpIX) concentration versus time plots for the placebo period of MUsT-1. **A**: Baseline-adjusted plasma concentrations of ALA [ng/mL] are plotted against time [h]. Values below 0 after baseline adjustment were set to zero for figures of PK concentration data. Baseline was calculated from one pre-dose value. Each line depicts one individual profile (n=12). The mean concentration versus time profile is depicted as solid line. **B**: Plasma concentrations of PpIX [ng/mL] are plotted against time [h]. As baseline-adjustment of PpIX generated an abundance of values <0, unadjusted data were chosen for presentation and corresponding baseline levels are indicated. Baseline was calculated from one pre-dose value. Each solid line depicts one individual profile (n=12), dashed line depicts geomean baseline level of PpIX in MUsT-1. The mean concentration versus time profile is depicted as solid line, with all BLQ values included as 0 in the calculation of the mean. ALA: 5-aminolevulinic acid, c: baseline-adjusted / unadjusted plasma concentration, MUsT: maximal usage pharmacokinetic trial, PDT: Photodynamic Therapy, PpIX: protoporphyrin IX.

**Supplementary Figure S1:**


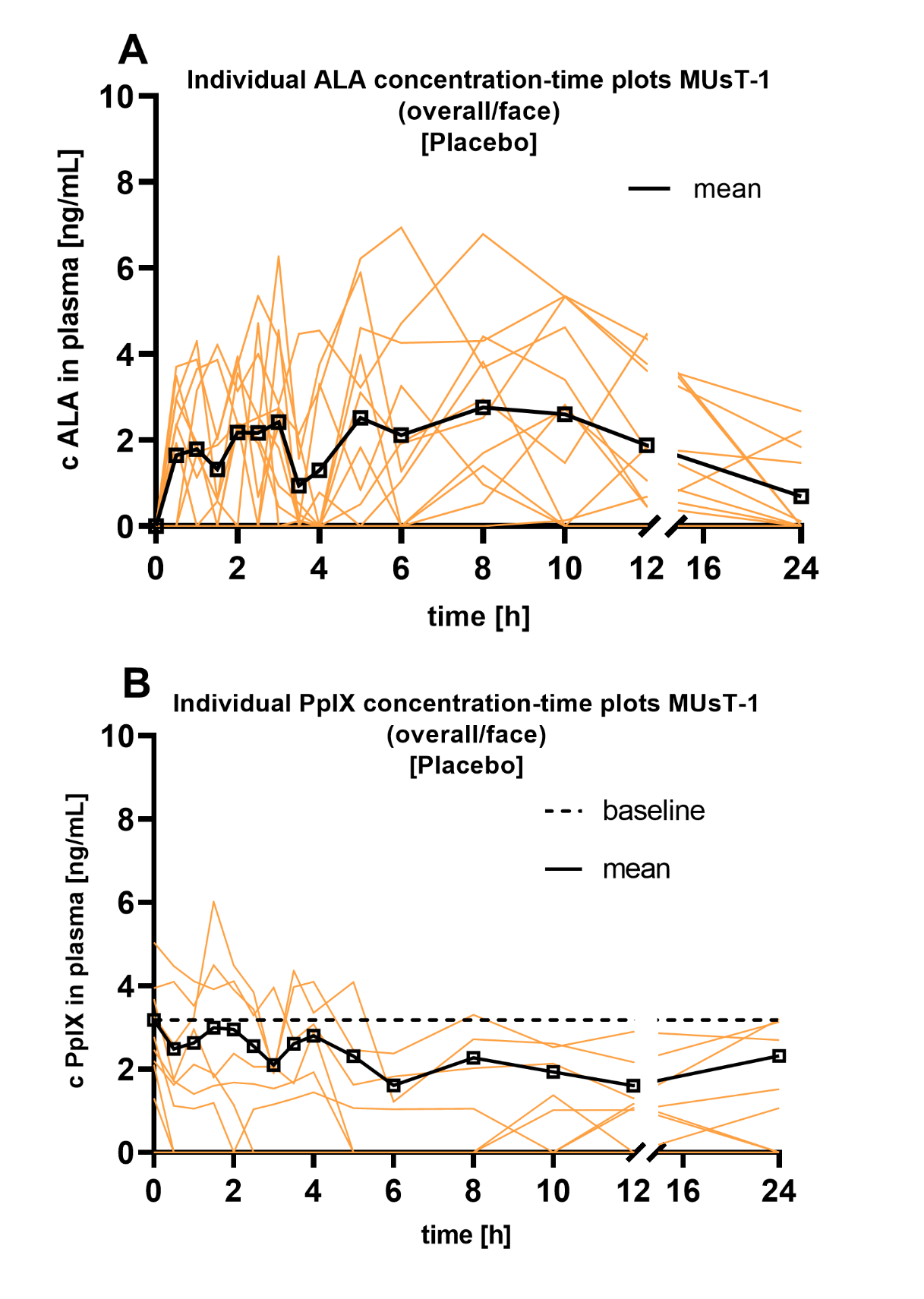

Supplement: Supplementary file 1 — SUPPLEMENTARY INFORMATION [file CPDD-11-535-s001.docx]
